# Sustainable scaling up of good quality health worker education for tuberculosis control in Indonesia: a case study

**DOI:** 10.1186/1478-4491-7-85

**Published:** 2009-11-16

**Authors:** Carmelia Basri, Karin Bergström, Wanda Walton, Asik Surya, Jan Voskens, Firdosi Metha

**Affiliations:** 1 National Tuberculosis Control Programme, Ministry of Health, Jakarta, Indonesia; 2 Tuberculosis Strategy and Operations, Stop TB Department, World Health Organization, Geneva, Switzerland; 3 Division of Tuberculosis Elimination, Centers for Disease Control and Prevention, Atlanta, GA, USA; 4 KNCV, The Hague, Netherlands; 5 World Health Organization, Jakarta, Indonesia

## Abstract

**Background:**

In 2000, an external review mission of the National Tuberculosis Control Programme of Indonesia identified suboptimal results of TB control activities. This led to a prioritization on human resource capacity building representing a major shift in the approach following the recommendations of the external review team.

**Case description:**

The National Tuberculosis Control Programme (NTP) used a systematic process to develop and implement two strategic action plans focussing on competence development based on specific job descriptions. The approach was a change from only focussing on training, to a broader, long term approach to human resource development for comprehensive TB control.

A structured plan for capacity building, including standardized competency based training modules and curricula, was developed in the first phase. This was supported by an organisational system comprised of a training focal point, master trainers, and regional training centres in which nationwide training of supervisors was implemented. Training was expanded to the health service delivery level in the second phase, as well as broadened in the scope of activities beyond training to also include other aspects of human resource development.

**Discussion and evaluation:**

The result was improved technical and managerial capacity of health workers for TB control at all levels. The impact on case detection and treatment outcome was spectacular, with major improvements in quality of all aspects of service delivery.

**Conclusion:**

The strategic decision by the NTP in 2000 to put the highest priority on capacity building has resulted in impressive progress towards TB control targets, a progress that despite many challenges has been sustained.

## Background

Indonesia ranks third in tuberculosis (TB) burden in the world with an estimated annual incidence of 239 cases per 100 000 people (108 sputum smear positive [ss+] cases per 100 000 people per year) and an estimated prevalence of 262 cases per 100 000 people [1]. DOTS, the TB control strategy recommended by the World Health Organization (WHO) [2], was introduced in Indonesia in 1992 with combined tuberculosis-leprosy pilot projects in four Sulawesi provinces (supported by KNCV Tuberculosis Foundation [KNCV] and Nederland's Leprosy Relief [NLR]). Initial trials achieved high cure and success rates.

In 1995, WHO declared a TB emergency in the country. This led to the adaptation of the DOTS strategy in 1995, with the gradual expansion to all provinces. This expansion however, resulted in inappropriate implementation in some areas with detrimental results to the quality of the programme.

In February 2000, a review of the National Tuberculosis Control Programme (NTP) of Indonesia was conducted with the assistance of WHO and KNCV. The review mission concluded that all five basic elements of the DOTS strategy appeared to be weak, except for the political commitment at national level, as demonstrated by the establishment of the Gerdunas (i.e., the national Stop TB Partnership) in 1999. Programme expansion had been too rapid and ambitious, without adequate preparation such as training and supervision of district staff. As a consequence, results for case detection were well below expectations. The case detection rate (CDR) for new sputum smear positive pulmonary TB was 19%, while the treatment success of these new sputum smear positive cases was 87.4%. The mission also concluded that many operational problems were caused by shortcomings in technical and management capacity at all levels of the health services, especially with regard to manpower, equipment, supervision, logistics, health information systems, and planning. This was due to suboptimal quality of training conducted in the past. Training was hampered by lack of planning, scant budget allocations, no standardized curricula, deficient training material, shortage of competent trainers, inadequate teaching methods and evaluations, as well as lack of follow-up. Problems were further complicated by the erratic distribution of anti-TB drugs since 1999, which led to the unavailability of drugs in many districts. A considerable backlog in training was identified; additionally, several provinces reported a problem of trained staff being transferred to other places.

With relation to capacity building, the review team recommended that the training backlog should be immediately addressed. This included a detailed analysis of the current training needs and development of a comprehensive training plan within the following 3 months. The team also recommended that attention to quality assurance should also be in place. The overall capacity at provincial and district levels in terms of manpower, supervision, and training should be strengthened. Following the review mission, the NTP granted capacity building the highest priority and radically revised methodologies and approaches used in their human resource development activities as part of gradual programme strengthening. We report on this strategic development of the human resources involved in the NTP in the period 2000-2006, following the recommendations of the review mission in 2000, and the results of these activities. It should be noted that this was not a pilot project, but part of routine programme management by the Ministry of Health.

## Case description

The systematic development of capacity in the NTP can be divided into two phases: The 2000 - 2002 "Plan to build capacity" and the 2003-2006 "HRD-TB strategic plan for the NTP, Indonesia". In the first phase, the focus was put on developing a structured system for providing training of high quality, both technically and educationally. In the second phase, the focus was on implementation, scaling-up, quality control, and addressing new human resources development (HRD) challenges as the progress towards TB control targets started accelerating.

### 2000-2002: Plan to build capacity

Following the review mission in 2000, the NTP undertook a problem analysis of the NTP capacity including rapid assessment of the human resources and training situation. The findings confirmed the observations made by the external review mission. Based on this, the NTP decided to do a complete restructuring of all training activities following strategic, competency based methodologies and accepted educational standards [3]. A comprehensive plan for capacity building in the NTP was developed for the period 2000-2002, followed by a project proposal for funding of the plan. The funding proposal was approved by the Dutch government. The overall goal of the plan was to improve the quality of the services delivered to TB patients through improvement of the skills of health workers at the various levels; a secondary goal was to improve the efficiency and cost-effectiveness of TB control programme management. To achieve this, it was also necessary to strengthen the intermediate and central levels of the program. It was expected that the range of interventions would improve the quality of performance at the service delivery level. The plan included the following areas of intervention (or methods) related to capacity building:

1. Development of a training plan outlining specific strategies, revised or updated job descriptions, and standardized training material and curricula.

2. Development of a national resource group ('master trainers') for the strengthening of management capacity.

3. Development of provincial/district TB management and training teams.

4. Training of health service units (UPK).

5. Training of hospital staff and private practitioners.

6. Inclusion of TB case management in pre-service training curricula.

#### 1. Development of a training plan outlining specific strategies, revised or updated job descriptions, and standardized training material and curricula

**Figure 1:**
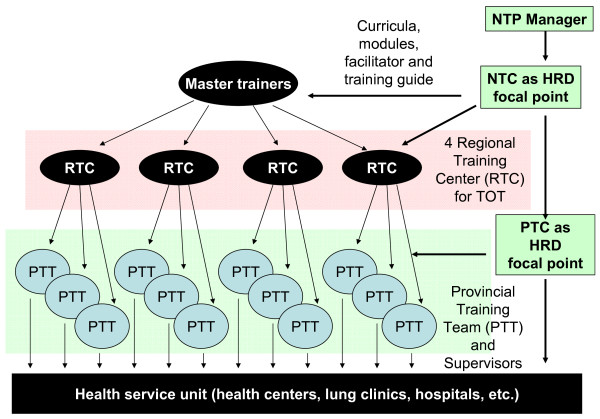
**Cascade training**. NTP: National Tuberculosis Control Programme. NTC: National training coordinator. PTC: Provincial training coordinator. HRD: Human resource development. TOT: Training of trainers.

The TB control central unit was strengthened by appointing additional staff sub-contracted with funding provided by the external donor. A training plan was developed. The priority was the rapid training of approximately 60% of supervisory staff at provincial and district levels. Approximately 850 supervisors were estimated in need of training. At service delivery level, the training need was even greater, with more than 7300 health centres with nurses and doctors involved in TB control. Given the training backlog, the high numbers of staff requiring training, and the urgent need of skilled staff, the approach used was stepwise cascade training (Figure 1). This model involved initial training of selected staff at central level, empowering them to become trainers themselves and to be actively involved in the training of staff in the following implementation tier; staff in each tier were then selected and capacitated to train staff in the implementation tiers below. Prior to training, a specific training curriculum for each level was developed. The NTP (supported by KNCV and WHO) reviewed the national TB guidelines and generated updated job descriptions for all staff involved in DOTS implementation. Job descriptions were based on the task analysis of activities required for appropriate DOTS implementation and were used to develop specific training curricula for staff at each level (master trainers, provincial and district supervisors, health centre doctors, nurses and laboratory technicians). The Central Unit of the NTP established a human resource working group representing all stakeholders. This group developed a set of ten competency based, basic modules for training provincial and district supervisors and staff at health centre level (Appendix 1). Additional modules were developed for training master trainers and course directors, as well as course facilitator guides. The course director and course facilitator guides included checklists to facilitate the planning and preparation of provincial and district training activities, and guidance for quality assurance of training courses. The training methodology included an ongoing assessment of participants through exercises, discussions, and observations.

#### 2. Development of a national resource group ("master trainers") for the strengthening of management capacity

The first step was the establishment of a master training committee. This committee was charged with the task of recruiting master trainers based on strict selection criteria, organizing the initial training course for master trainers, following-up on activities after this training (including regional planning meetings), and advising on the selection of four Regional Training Coordinators leading the regional teams of master trainers. Twenty-nine individuals from all over the country, including 23 individuals from the provinces and six from hospital settings (lung clinics) were identified and trained in the first training course for master trainers. The course included the full set of basic TB modules and lasted nine weeks. this included three weeks for the modules, four weeks for field exercises and assessment of training needs, one week for training skills development, and a final week for planning of regional trainings and other important health issues related to TB, such as HIV and leprosy. Training methodologies were based on competency development methods and methodologies; it included active participation methods, problem-based learning, and motivation techniques. Twenty-six trainers completed the course; from this group, four Regional Training Coordinators and a National Training Coordinator were selected and appointed. Master trainers became full time NTP employees and were posted to the four regional training centres covering all 30 provinces (Table 1), with five to seven master trainers per training centre.

**Table 1:** Regional training centres

Centre	Region	Number of Master Trainer	Provinces covered°
Padang	West Sumatra	6	8 provinces in the west
Makasar	South Sulawesi	5	10 provinces in the north and east
Murna Jati	East Java	6	6 central provinces
Ciloto	West Java	7	6 provinces in the west

#### 3. Development of provincial/district TB management and training teams

From April 2001 to February 2002, master trainers at the four regional training centres worked full time to train provincial and district supervisors. Approximately 25 supervisors were enrolled in each training course, divided in four to five groups per batch, keeping the targeted ratio of five trainees per trainer. The curriculum included all ten core training modules (Appendix 1), supplemented with field visits and practical work The training lasted for 14 working days. Master trainers established criteria for the selection of provincial and district trainers from the trained supervisors. Selection was made in coordination with regional master trainers and local health authorities. Provincial and district trainers were trained together, and became the teams that trained the staff from the health service units (UPK) and hospitals. Master trainers were continuously involved in the training of district staff; however, they gradually delegated training to provincial and district staff while providing direct supervision to ensure quality of district training.

The location of provincial/district training was selected carefully, to ensure it occurred in areas where the DOTS strategy had been implemented well. These districts served as 'centres of excellence', becoming examples for other districts. After successful completion of the course, the trainees received a course certificate. Trainees who were identified as potential trainers received a special certificate stating their potential of becoming a trainer for health centre level staff.

#### 4. Training of health centre level staff (UPK)

Training for staff at the health centre level started in March 2002. The courses for doctors and nurses took five working days to complete and included six training modules (modules 1 and 2, parts of module 3, and modules 4, 6 and 8). Training for laboratory technicians took nine days and included four modules (selected parts of modules 1 and 2, module 3 and parts of module 9). Post training evaluation was carried out at health centre level during regular supervisory visits to monitor the quality of the training process, identify shortcomings, and to provide feed back to the regional training centre.

#### 5. Training of hospital staff and private practitioners

The initial assessment had identified hospital staff and private practitioners as a key target group for capacity development for the implementation of the DOTS strategy. According to the plan, provincial/district training teams were going to train hospital doctors and nurses, together with private practitioners, in two-day seminars. To disseminate knowledge and skills, trained health unit doctors were to organize one day 'micro symposia' on TB/leprosy and HIV for all non designated staff in their health service units. Hospital managers were to be invited for one-day seminars to disseminate and discuss NTP guidelines. However, it was found that training curriculum and materials needed adaptation to suit the needs for these specific providers. In particular, the specific characteristics of hospital set-up and referral mechanisms between these providers and general health services needed to be covered in the training program.

#### 6. Inclusion of TB case management in pre-service training curricula

The plan included activities to initiate the process to update curricula in national and private training institutions, to be in line with national guidelines for TB control, to ensure the long term sustainability of competences for all health workers involved in TB control. Co-ordination between the Gerdunas (i.e., the National Stop TB Partnership) and the national government training institutions was to be improved through seminars at provincial level, starting in 2001. Gerdunas were to produce and disseminate the NTP case management guidelines to the training institutions, which were expected to incorporate the NTP case management guidelines into their curricula. However, activities were delayed and in the 2000-2002 period only a first workshop was held with representatives of medical training schools.

### 2002-2006: HRD-TB strategic plan for the NTP Indonesia

The HRD-TB strategic plan was developed as a continuation of the 2-year capacity building plan, built on the foundation and results of this first plan, and was part of the NTP's overall 5-year strategic plan for DOTS expansion. Funding for this plan was initially made available through United States Agency for International Development (USAID) and Canadian International Development Agency (CIDA). Later funding was through the Global Fund for Aids, Tuberculosis and Malaria (GFATM, now GF). A long term approach was taken to training and other activities for human resource development. This represented a shift from a 'training only' focus to a broader strategic approach to HRD, thus representing a paradigm shift within the NTP [4]. This also represented a shift towards a stronger role for long term, comprehensive management of HRD at all levels.

The overall strategic goal in the plan for HRD was to ensure that all staff involved in TB control at all levels had the appropriate skills, and that there was enough staff at the right time, to support programme implementation to reach the TB control goals of the programme. Operational policies and strategies were developed to achieve this goal; specific targets were set with regards to (i) availability of trained staff for TB control at the health centre, district and provincial level; (ii) availability of trained staff for TB control for hospitals; and (iii) the development of a TB component for the curricula in basic training institutions. The key activities listed in the plan were: continue to develop and revise (as necessary) training guidelines, curricula and modules for both pre- and in-service training and education; conduct training needs assessments; ensure annual planning for TB-HRD at all levels; supervise and monitor the implementation for these plans; and develop an information system for monitoring the availability of trained staff at various levels.

## Discussion and evaluation

The results are presented in two sections: the first section describes the capacity development activities towards the goal as outlined in the HRD strategic plan; the second section describes the impact of the capacity development activities on progress towards national and global TB control targets (5,6)

### Capacity development

**Figure 2:**
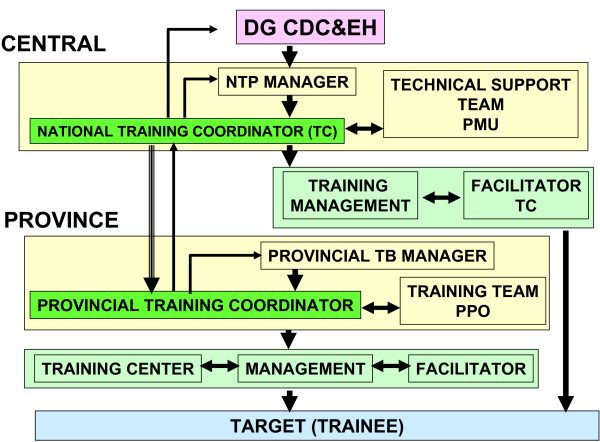
**Training organization**. DG CDC & EH: Director-general, Disease Control and Environmental Health. NTP: National Tuberculosis Control Programme. PMU: Programme Management Unit. PPO: Provincial Project Officer.

A structured training system with standardized, competency based training modules, curricula, master trainers, and regional training centres was developed (Figure 2). A total of 991 provincial and district supervisors were trained during the first 15 months of the implementation of the training activities. Due to staff turnover (attrition as a consequence of de-concentration and health sector reforms implemented in 2001) and some confusion regarding selection criteria, more than one supervisor per district and province were trained. In 2003, training was provided for 333 recently appointed supervisors, all of them filling positions left by previously trained supervisors who were transferred to work in other positions outside the TB control program.

Training of doctors, nurses, and laboratory technicians at health centre level started in March 2002. By the end of 2002, training had been provided for about 35% of the doctors and nurses (2500), and for 38% (1000) of the laboratory technicians. The training of health centre staff was delayed due to organizational limitations during rapid scaling up of training. Staff turnover of around 10-20% per year further slowed down the efforts to increase the availability of competent staff at this level.

**Figure 3:**
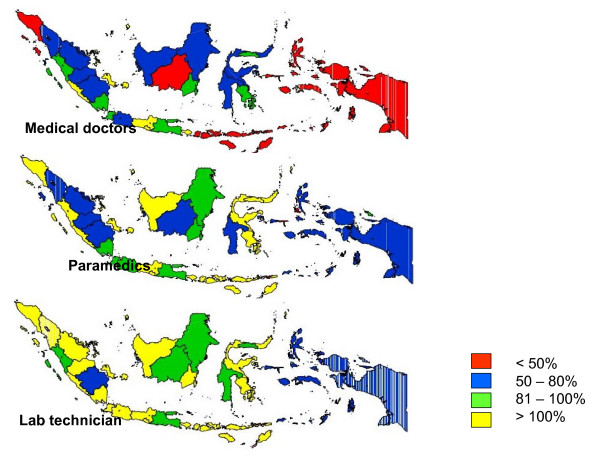
**Percentage of health center staff trained to standard, by province, Indonesia, mid 2005**. % Trained to standard.

By late 2004, an information system, including staffing standards, had been established that allowed the monitoring of availability of trained staff by health facility, especially at health centre level (Figure 3). In 2003, a full-time National Training Coordinator (NTC) was appointed at the central level of the NTP, Additionally, terms of reference were developed to strengthen the management of training activities and co-ordinate activities nationally. In the same year, 30 Provincial Training Coordinators (PTC) were appointed. However, due to the 'zero growth' policy of the Ministry of Health, this did not represent new appointments but rather the creation of an additional role to previously employed staff or the recognition of previously *ad hoc *performed activities. The role of the PTC was to plan, organise, and monitor training at province level in the efforts to accelerate capacity building. The central team was further strengthened with additional contracted staff; and at provincial level, Provincial Project Officers (PPO) were appointed.

In 2004, post-graduate training began for senior program staff through the advanced course for supervisors on DOTS acceleration (ACDA) to further strengthen technical and managerial skills of selected central staff and provincial supervisors. This course was a modification of an international training course curriculum adapted to the Indonesian situation.

**Figure 4:**
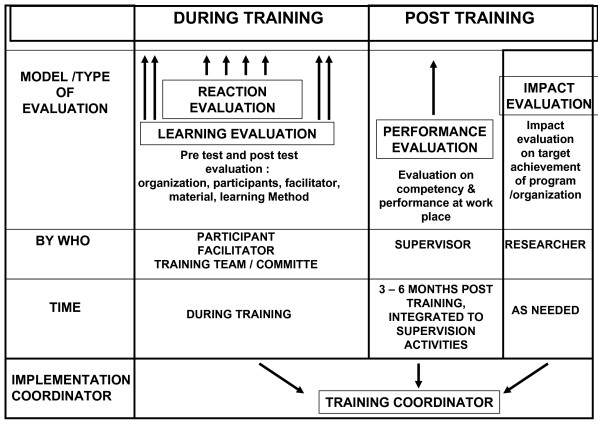
**Methods for training evaluation**.

A system was developed to strengthen training evaluation to further improve quality (Figure 4). Involvement of the lung clinics started with sensitization of managers, followed by a one-week training of medical staff. Gradually, the involvement further expanded to include both public and private hospitals. By 2004, all 34 lung hospitals and lung clinics in the country, in addition to an estimated 20% of all public and private hospitals, had become involved in the national DOTS program.

A new competency-based curriculum for medical schools was developed; introduction in medical faculties was started in a phased manner after extensive consultations. Introduction of DOTS in nursing schools started in 2003, and is now being implemented in 18 schools.

### Progress towards TB control targets

**Figure 5:**
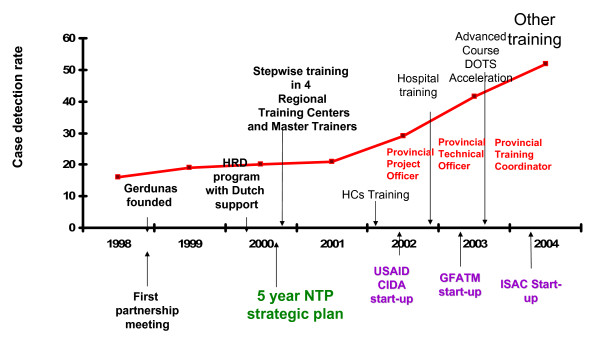
**DOTS expansion and TB training**. USAID: United States Agency for International Development. CIDA: Canadian International Development Agency. HCs: GFATM: Global Fund for Aids, Tuberculosis and Malaria (now GF). ISAC: Intensified support and action in countries. NTP: National Tuberculosis Control Programme. United States Agency for International Development (USAID) and Canadian International Development Agency (CIDA). Later funding was through the Global Fund for Aids, Tuberculosis and Malaria (GFATM, now GF).

**Figure 6:**
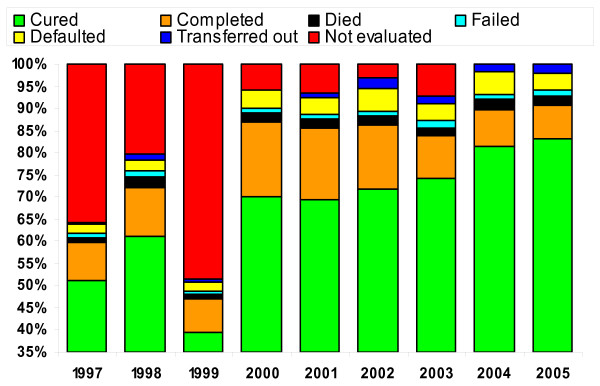
**Treatment outcome (1997-2005)**.

A rapid increase in case detection rates was observed in provinces after the training of health centre staff, whereas neighbouring provinces with low levels of training showed slow progress. This increase took place before external donor support (USAID, CIDA, GFATM) was stepped up. Case notification of all types of TB and new smear positive pulmonary TB increased from 84 591 to 285 030, and from 52 338 to 174 953 respectively in the period 2000-2006 (Table 2) [5,6]. This represented an increase in the case detection rate for new smear positive TB from 19% in 2000, to 54% in 2004 and 76% in 2006 (Figure 5) [5]. Treatment success rates were sustained at over 85% over the same period, and achieved 89% success for the 2004 cohort [5]. Major improvements were also made in each category of treatment outcome, representing a major quality improvement in programme implementation (Figure 6).

**Table 2:** Number of TB cases notified, 1997-2004

	Pulmonary tuberculosis				
			
	Smear-positive				
				
Year	New	Relapse	Smear-negative	Extra Pulmonary tuberculosis	Total
					

1997	25 420	497	3125	4174	33 214

1998	29 781	1177	8217	382	39 557

1999	49 333	2205	2792	1105	73 309

2000	52 338	2478	28 225	1550	84 591

2001	53 965	2708	34 547	1818	92 792

2002	76 230	3731	72 219	3008	155 188

2003	92 566	4086	77 561	4047	178 260

2004	128 913	4421	76 961	4266	211 753

2005	158 632	4442	90 447	6137	259 658

2006	174 953	4197	90 818	7020	285 030

## Discussion

During the period of 2000 - 2006, remarkable progress was made in building technical and managerial capacity for TB control at all levels within the health system. This had a direct effect on program performance, particularly on all aspects of TB case management, TB case notification, and the quality of surveillance. Management for HRD at the central level, and various other levels of the health system, was strengthened with the responsibilities for HRD clearly established. This illustrates the importance accorded to HRD activities within the NTP.

Much effort was put into building a management system for capacity building, the training of the national master trainers, and the development of the basic training modules, thereby constructing a solid basis for subsequent expansion. At health centre level, there was a significant improvement of skills in DOTS implementation. This included laboratory performance and compliance with NTP guidelines. However, the high staff turnover, especially at the health centre level, complicated and delayed progress. Furthermore, at the health centre level, the workload for nurses is often high due to tasks and responsibilities other than TB control. The training of district and provincial supervisors led to improved supervision which contributed to improved motivation and performance. These regular supervisory visits, including, but not limited to, data collection are key to quality improvement and sustainability.

A key factor to the success was the appointment of a focal person for HRD, the NTC, within central level NTP, as well as the systematic approach to establishing an organizational structure at all levels for HRD and the standardization of training materials and procedures. The importance of having the HRD focal person located at the central unit to facilitate close collaboration with all members of the central unit was highlighted. Regular review meetings between the NTC and the PTCs are essential to support staff motivation and to ensure sustained development. Technical assistance - short term and long term - was an additional strong contributing factor to the success. In addition, earmarked financial resources for specified aspects of HRD, as well as for overall programme implementation, gradually increased, thereby facilitating the step-wise process of implementing the plans. One of the biggest constraints faced in the implementation has been related to organizational limitations and bureaucracy, which led to delayed disbursement of funds and subsequent delays in implementation.

There has been significant improvement in staffing at central and provincial level due to contracting of additional staff, made possible through donor funding. However understaffing is still a problem at provincial and district level; there is still a relative shortage of supervisors to enable regular and constructive supervision and data collection, especially with regard to supervising the large number of hospitals. In addition, provincial and district level supervisors also often function as training course facilitators. This leads to a heavy workload with implications on the quality of training and the frequency and quality of supervision.

Indonesia is a large country and the challenges in implementation differ from province to province. The capacity for program management, including HRD management capacity, is weak in some provinces and some PTC's are weak. Linking hospitals to the national DOTS program is a major challenge due to the large number of hospitals (>1200) and the large number of staff who need to be trained; the characteristics of the target group (e.g., specialists reluctant to follow DOTS guidelines); and the specific issues related to DOTS implementation in hospitals. Though several basic training institutions have included DOTS in the basic curricula, the majority have not.

The management information system to monitor the availability of competent staff at the health facility level still needs to be simplified and optimized at all levels. The post training evaluation system is still not used optimally, as supervisors in some areas have suboptimal supervisory skills. And since the NTP is expanding its activities in TB control, shifting from basic DOTS implementation to the new, more comprehensive Stop TB Strategy [7], new training needs are emerging (e.g., drug management, patient education, advocacy, TB/HIV, management of multidrug-resistant TB, and the use of electronic registers).

## Conclusion

As the implementation of the DOTS strategy progresses, the complexity of HRD increases, with major challenges related to the long-term management of training and staffing remaining. However, the mechanism of in-depth assessments through comprehensive monitoring missions, including key internal and external partners, has been continued following the mission in 2000; and these assessments do include the HRD activities. This mechanism provides a systematic, regular situation analysis that includes identification of problems. This enables the TB control program to identify and implement appropriate solutions in a consistent manner.

HRD for any service delivery area is a complex and long term undertaking; the experience of the NTP shows that HRD issues get more complex as the programme develops and expands, thereby adding to the already substantial HRD needs. The strategic decision by the NTP in 2000 to put the highest priority on capacity building has resulted in impressive progress towards global TB control targets - a progress that has been sustained despite many challenges. It is also clear that without the substantial amounts of external funding that were gradually made available to the NTP, this progress would not have been possible. However, equally important to the success and sustainability is the continuously strengthened management capacity at all levels. Ensuring that all staff involved are highly competent, as well as ensuring that there are enough staff available, requires continued priority attention to training and staffing activities from the NTP, other sectors of the Ministry of Health, other ministries, as well as from donors and other partners, over the coming years.

The 2002-2006 strategic plan for HRD was not only the first HRD plan for the NTP in Indonesia, it was also the first comprehensive strategic plan for HRD in any NTP. The experiences gained by the NTP in Indonesia, in collaboration with key partners, are major contributing factors in the development of global strategies for HRD in TB control.

## Competing interests

The authors declare that they have no competing interests.

## Authors' contributions

CB and AS were responsible for the implementation of the work of the NTP as described in this article. JV, FM and KB have provided ongoing technical assistance. CB, KB and WW drafted the manuscript. All authors read and approved the final manuscript.

## Appendix

### Appendix 1

1. Programme

2. Case finding

3. Laboratory activities

4. Treatment

5. Recording and reporting

6. Monitoring and evaluation

7. Supervision

8. Health Promotion

9. Logistics

10. Planning
